# Polyketone-Based Anion-Exchange Membranes for Alkaline Water Electrolysis

**DOI:** 10.3390/polym15092027

**Published:** 2023-04-25

**Authors:** Ottavia Racchi, Rebecca Baldassari, Esteban Araya-Hermosilla, Virgilio Mattoli, Pierpaolo Minei, Alfonso Pozio, Andrea Pucci

**Affiliations:** 1 Dipartimento di Chimica e Chimica Industriale, Università di Pisa, Via Moruzzi, 13, 56124 Pisa, Italy; 2 Center for Materials Interfaces @SSSA, Istituto Italiano di Tecnologia, Viale Rinaldo Piaggio, 34, 56025 Pontedera, Italyvirgilio.mattoli@iit.it (V.M.); 3 SPIN-PET, Via R Piaggio, 32, 56025 Pontedera, Italy; minei@spinpet.it; 4 ENEA CR Casaccia, Via Anguillarese, 301, 00123 Rome, Italy; alfonso.pozio@enea.it; 5 CISUP, Centro per l’Integrazione della Strumentazione dell’Università di Pisa, Lungarno Pacinotti, 43, 56126 Pisa, Italy

**Keywords:** water electrolysis, anion-exchange membrane, polyketone, Paal–Knorr, membranes, quaternary ammonium

## Abstract

Anion-exchange membranes (AEMs) are involved in a wide range of applications, including fuel cells and water electrolysis. A straightforward method for the preparation of efficient AEMs consists of polymer functionalization with robust anion-exchange sites. In this work, an aliphatic polyketone was functionalized with 1-(3-aminopropyl)imidazole through the Paal–Knorr reaction, with a carbonyl (C_CO_ %) conversion of 33%. The anion-exchange groups were generated by the imidazole quaternization by using two different types of alkyl halides, i.e., 1,4-iodobutane and 1-iodobutane, with the aim of modulating the degree of crosslinking of the derived membrane. All of the membranes were amorphous (T_g_ ∼ 30 °C), thermally resistant up to 130 °C, and had a minimum Young’s modulus of 372 ± 30 MPa and a maximum of 86 ± 5 % for the elongation at break for the least-crosslinked system. The ionic conductivity of the AEMs was determined at 25 °C by electrochemical impedance spectroscopy (EIS), with a maximum of 9.69 mS/cm, i.e., comparable with that of 9.66 mS/cm measured using a commercially available AEM (Fumasep-PK-130). Future efforts will be directed toward increasing the robustness of these PK-based AEMs to meet all the requirements needed for their application in electrolytic cells.

## 1. Introduction

Anion-exchange membranes (AEMs) have recently been developed for application in electrochemical systems [1]. AEMs consist of polymers with positively charged functional pendant groups. Examples of starting polymers to fabricate AEMs include polysulfones, polystyrene and divinylbenzene or butadiene copolymers, polyethylene oxides, polychloroprenes, and polyketones (PKs) [2,3,4,5,6,7,8]. These membranes are characterized by the presence of positively charged groups, such as -NH_3_^+^, -NRH_2_^+^, -NR_2_H^+^, -NR_3_^+^, and -PR_3_^+^ [9,10]. These novel membranes offer advantages compared with proton-exchange membranes (PEMs) in electrolysis, alkaline electrolysis, and even in fuel cells [11], since they do not require the use of noble metals as catalysts and use only distilled water or a low-concentration alkaline solution as an electrolyte instead of concentrated KOH [12]. All these advantages result in much cheaper technology, triggering the increasing demand to develop AEMs with higher chemical stability and conductivity [10]. AEMs must meet certain mechanical, chemical, and thermal requirements. They must ensure efficient transfer of hydroxyl groups from one electrode to another, so as to ensure high ionic conductivity and limit gas crossover, as their diffusion would lead to lower cell efficiency and performance. Moreover, AEMs must be chemically stable, as they work in strongly basic environments. Ideally, they should be long-lasting and not dissolve in an aqueous environment but be easily processable in organic solvents. All of the above properties must be retained at the working temperature of the cell [10,13]. To date, no membrane has presented all of these parameters together, and investigations have been focused on efficient AEMs with the required chemical and thermal stability [14]. In this sense, the membrane’s chemical and thermal performances depend strongly on the nature of the moiety capable of carrying hydroxyl anions and the polymer backbone [15]. Among the different cationic groups, the quaternary ammonium groups exhibit greater chemical and thermal stability than the quaternary phosphorus or tertiary sulfur groups [13]. Nevertheless, apart from temperature, the main cause of cationic groups’ degradation when used as anion-exchange sites is the basicity of the environment, which activates adverse chemical reactions such as the Hofmann elimination [16]. Therefore, the main challenge is the development of an AEM with OH^−^ ion conductivity comparable with that of H^+^ in a PEM, flanked by durable anion-exchange sites [17]. 

This work aimed to prepare imidazole-functionalized polyketone-based membranes for AEM water electrolysis. Synthesized from industrial waste gas (carbon monoxide) and olefin as raw materials, aliphatic polyketones have the great advantage of being highly reactive towards nucleophilic addition on the 1,4-dicarbonyl groups, which facilitates the conversion of PKs into polymers containing various chemical functional compounds, such as alcohols, ketals, thiols, and pyrroles [18,19]. PKs also have excellent mechanical properties, including pressure and heat resistance [20]. Notably, the reaction of the 1,4-dicarbonyl units of PK with primary amine leads to the formation of N-substituted pyrrole units through the Paal–Knorr reaction [21]. This reaction occurs without using any solvents and has the advantage of having only water as a secondary product [22,23]. The functionalization of PKs with imidazole groups allows the introduction of quaternary ammonium cation groups with two different types of haloalkanes. 1-Iodobutane was used as a quaternizing molecule for the grafted imidazole moieties, whereas 1,4-iodobutane also acted as a crosslinking agent. These two halides were used in different quantities to modulate the crosslinking degree of the polymer and its mechanical properties. 

## 2. Experimental Section

### 2.1. Materials

1-(3-Aminopropyl)imidazole (Sigma-Aldrich, Milan, Italy), 2,5-hexandione (Sigma-Aldrich, Milan, Italy), 1,4-diiodiobutane (Sigma-Aldrich, Milan, Italy), and 1-iodiobutane (Sigma-Aldrich, Milan, Italy) were used as received. Aliphatic polyketones made of ethylene, propylene, and carbon monoxide were synthesized according to a previously reported procedure, yielding a polyketone with 30 mol% ethylene and 70 mol% propylene (PK30, Mw 2930) [24]. 1,1,1,3,3,3-Hexafluoro-2-propanol (HFIP) (abcr), dichloromethane (Sigma-Aldrich, Milan, Italy), methanol (HPLC-grade, Carlo Erba, Milan, Italy), chloroform (HPLC-grade, Carlo Erba), acetone (Sigma-Aldrich), hexane (Sigma-Aldrich, Milan, Italy), 0.1N HCl standard solution (abcr), 0.1N NaOH standard solution, chloroform-d (Sigma-Aldrich, Milan, Italy), and dimethyl sulfoxide-d (Sigma-Aldrich, Milan, Italy) were used as received.

### 2.2. Preparation of the Anion-Exchange Membranes

#### 2.2.1. Polyketone Functionalization with 1-(3-aminopropyl)imidazole

**Figure 1:**
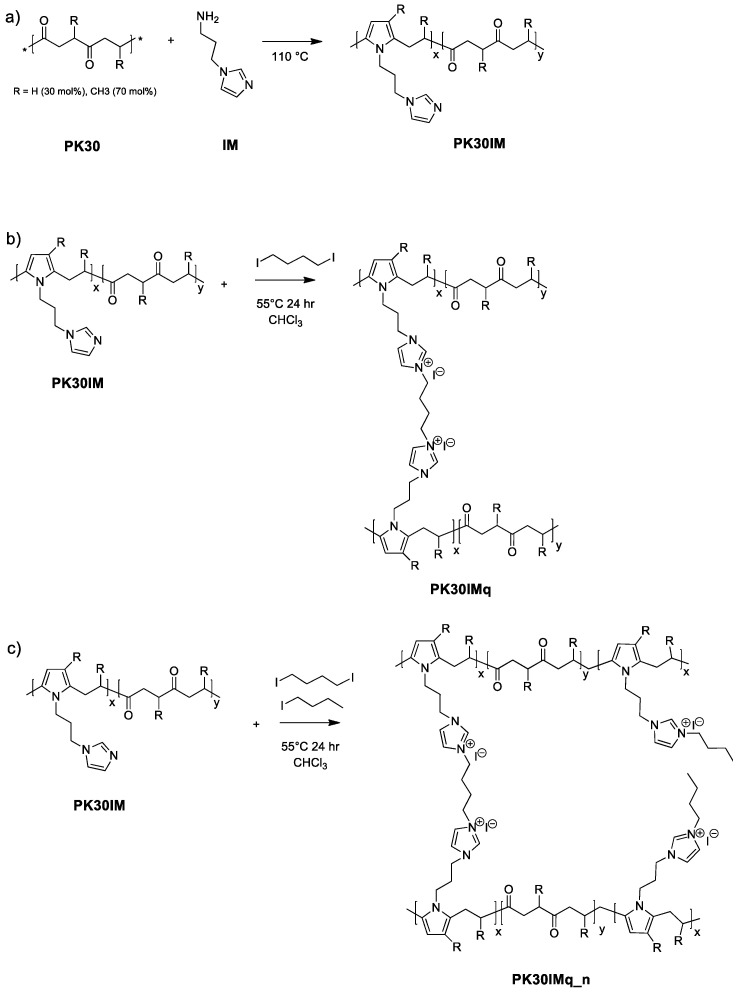
(**a**) Synthesis of PK30IM; R represents -H (30 mol%) or -CH_3_ groups (70 mol%). (**b**) Synthesis of the quaternized imidazole-functionalized polyketone PK30IMq. (**c**) Synthesis of the quaternized and crosslinked PK30IMq_n, with n = 80%, 90, or 95% of quaternized imidazole moieties.

PK30 was functionalized with 1-(3-aminopropyl)imidazole (PK30IM, Figure 1a) to reach nearly 35% of polyketone’s dicarbonyl group conversion (Table 1). First, 12.28 g of PK30 was placed in a 100 mL round-bottomed flask equipped with a mechanical stirrer, a reflux condenser, and a dripping funnel. Then, 4.51 g of 1-(3-aminopropyl)imidazole was added to the polymer, and the reaction was carried out for 3 h at 110 °C. The product was dissolved with chloroform and purified by solvent extraction with brine. The process was repeated three times to remove any unreacted amine, and the organic solvent was removed by evaporation. The carbonyl conversion (C_CO_)—i.e., the molar fraction of 1,4-dicarbonyl units reacted—was determined by elemental analysis (Table S1) using the following equation [25,26]:

(1) $$C_{CO}=\frac{N\times M_{c}}{n\times M_{N}+N\times \left(M_{c}-M_{r}\right)}$$

where *N* is the nitrogen content per gram obtained by elemental analysis, *M_N_* (g/mol) is the atomic mass of nitrogen, *n* is the number of nitrogen atoms in the N-substituted pyrrole units (3 in this case), *M_c_* (g/mol) is the molecular weight of the 1,4-dicarbonyl in the PK30 backbone (131.6 g/mol for PK30), and *M_r_* is the molecular weight of a converted 1,4-dicarbonyl unit (220.6 g/mol for PK30IM).

#### 2.2.2. Membrane Preparation

Next, 0.5 g of PK30IM was dissolved in 20 mL of chloroform and stirred for 20 min at room temperature, and the appropriate amount of 1-iodobutane and/or 1,4-iodobutane was added to the solution to quaternize the PK30IM imidazole groups (Figure 1b). The solution was then poured into a Teflon Petri dish, and a homogeneous membrane with an average thickness of 150 μm was obtained after solvent evaporation and drying at 50 °C for 24 h. The membranes were washed with hexane to remove the unreacted quaternizing agents and then dried under a hood. Specifically, based on the elemental analysis (Table S1), the quantities of the halides added to the mix were calculated by the following formulae:

(2) $$0.5\;g\;of\;PKIM*\;C_{co}=mol_{N}$$

(3) $$g_{1,4-iodobutane}=\frac{mol_{N}*MW_{1,4-iodobutane}}{2}$$

(4) $$g_{1-iodobutane}=mol_{N}*MW_{1-iodobutane}$$

where *C_CO_* corresponds to the molar fraction of 1,4-dicarbonyl units reacted via the Paal–Knorr reaction, *MW_1,4-iodobutane_* is the molecular weight of 1,4-iodobutane, and *MW*_1-_*_iodobutane_* is the molecular weight of 1-iodobutane.

### 2.3. Instruments and Methods

FTIR-ATR spectra were recorded using a Nicolet iS50 FTIR-ATR spectrometer. The solid samples were analyzed in ATR mode by setting 32 scans and a resolution of 4 cm^−^^1^. The samples were put in the oven at 50 °C for 24 h to eliminate water traces. The ^1^H-NMR spectra were recorded at room temperature on a JEOL spectrometer operating at 500 MHz and using 5 mm tubes, setting 32 scans. The samples were prepared by dissolving 20 mg of sample in 0.7 mL of (CD_3_)_2_SO. The NMR spectra were registered at 25 °C, and the chemical shifts were assigned in ppm using the solvent signal as a reference. Differential scanning calorimetry (DSC) was performed with a TA Instruments Discovery DSC 250 calorimeter. The samples were prepared by inserting 3–10 mg of the product into a 3 mm diameter aluminum Tzero pan and closed with a Tzero hermetic lid.

The thermal stability of the samples was evaluated by thermogravimetric analysis (TGA) with a TA Instruments TGA Q500. The experiments were carried out from 30 to 600 °C at 10 °C/min and under a nitrogen flux (60 mL/min). The elemental composition of the polymers was analyzed using an Elementar vario micro cube for nitrogen, carbon, and hydrogen. The scanning electron microscopy (SEM) analysis was performed with a JEOL 5600-LV microscope equipped with an Oxford energy-dispersive X-ray spectroscopy (EDS) microprobe. The tensile properties were measured with a Tinius Olsen H10KT dynamometer equipped with a 500 N load cell and according to the ASTM D638-10, with a 5 mm/min extension rate.

### 2.4. Characterization of the Anion-Exchange Membranes 

#### 2.4.1. Water Uptake (WU)

The membrane was activated in 1 M KOH at 50 °C for 30 min in order to exchange the counter anions into the OH^−^ form. Then, the membrane was immersed for another 30 min in degassed water. The excess water was removed from the membrane surface using a humidified filter paper. The WU values were calculated using the following equation:

(5) $$WU\;\left(\%\right)=\frac{W_{wet}-W_{dry}}{W_{dry}}\times 100$$

From the average of at least five measurements, the value of water uptake at the temperature of 50 °C was calculated.

#### 2.4.2. Ion-Exchange Capacity (IEC)

For the determination of the IEC (ion-exchange capacity), the membrane was first conditioned in a solution of 1 M KOH for 24 h. Then, it was washed with degassed water and immersed in 20 mL of a 0.1 M HCl solution for 24 h to neutralize all of the OH^−^ of the polymer. After removing the membrane from the HCl solution and carefully rinsing to collect all of the acid, the excess HCl was then titrated with a 0.1 M NaOH solution. The endpoint was determined by visual or potentiometric methods. The IEC was calculated as the mean of at least three measurements and expressed in milliequivalents per gram of dry membrane, according to Equation (6):

(6) $$IEC\;\left(meq/g\right)=\frac{meq_{HCl}-meq_{NaOH}}{g_{dry}}$$

### 2.5. Electrolytic Cell Tests

An appropriately sized sample of the quaternized membrane was used to prepare an MEA (membrane electrode assembly) and tested in an alkaline membrane water electrolysis test cell. The membranes were mounted in the test cell with GDEs (gas diffusion electrodes) made from a commercial fiber metal sintered steel AISI 316-L (Bekaert) with a thickness of 0.51 mm, a porosity of 82%, and a diameter of 16 mm. The anodic sides were supplied with a 0.5 M KOH solution contained in a 500 mL polyethylene tank using a dosing pump (KMS) with a flow rate of 100 mL/min. The output of the cathodic side was connected to a volumetric system for measuring the hydrogen produced. Characteristic E vs. i curves were recorded on the cell at room temperature (25 °C) in the current range 0–800 mA/cm^−2^, using a 1287 potentiostat/galvanostat (Solartron). Electrochemical impedance spectroscopy (EIS) measurements were performed in the 20 kHz–1 Hz frequency range at open-circuit voltage (OCV) with a 10 mV amplitude of the alternating signal, using a frequency response analyzer 1260 (Solartron). The EIS impedance spectra can be represented with Nyquist diagrams, in which the high-frequency intercept (*R_HF_*) represents the ohmic resistance of the system. The measured resistance value *R_HF_* is that relating to the high-frequency resistance, i.e., the intercept with the Z_Re_ axis by Z_Imm_ = 0 in the Nyquist diagram. Once this value is known, it is possible to calculate the surface-specific resistance *ASR* (Ω cm^2^) for the cell, taking into consideration the surface area of the membrane (2.0 cm^2^) in the electrolyzer. This measurement also allows estimation of the intrinsic conductivity of the membrane (i.e., the conductivity induced by counterions associated with positive ionic sites of the ionomer and favored by water molecules).

The samples were placed in the cell at 25 °C, and the conductivity was measured three times for each sample. From the value of the *R_HF_* of three measurements it is possible, knowing the thickness (*d*) and membrane area (*S*), to calculate the surface-specific resistance (*ASR*) and estimate the conductivity (σ) through the following two formulae:

(7) $$\;ASR=R_{HF}\times S\;$$

(8) $$\sigma =\frac{1}{R_{HF}}\frac{d}{S}=\frac{d}{ASR}\;$$

## 3. Results and Discussion

### 3.1. Preparation of the PK30IMq and PK30IMq_n 

We successfully functionalized PK30 with 1-(3-aminopropyl)imidazole through the Paal–Knorr reaction to produce a polyketone with imidazole pendant groups—PK30IM (Figure 1a)—with a carbonyl conversion (C_CO_) of 33%, as determined by elemental analysis (Table S1). Then, the imidazole groups of PK30IM were quaternized with 1-iodobutane and/or 1,4-iodobutane (see Table 1 and Figure 1b,c) to prepare the anion-exchange membranes (AEMs). Four membranes were obtained, i.e., PK30IMq, with 100% of the imidazole groups quaternized with 1,4-iodobutane, and PK30IMq_n (with n = 95, 90, and 80), with the imidazole groups converted by using progressive amounts of 1-iodobutane (i.e., 5, 10, and 20%) in the quaternizing mixture. The different compositions of the quaternizing agents were introduced in order to maintain a constant overall number of ion-exchange sites while changing the crosslinking degree of the polymer. 

**Figure 2:**
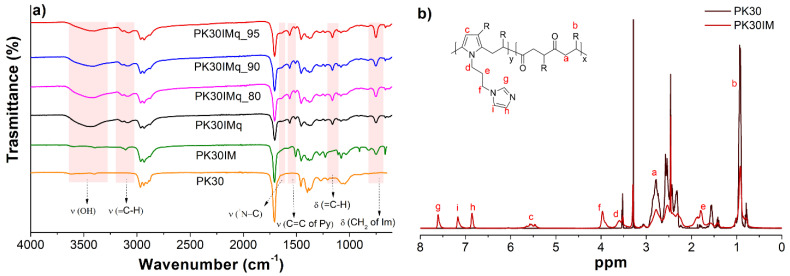
(**a**) FTIR spectra of PK30, PK30IM, PK30IMq, and PK30IMq_n (n = 80, 90, and 95) characterized in ATR mode. (**b**) ^1^H-NMR spectra of samples PK30 (**left**) and PK30IM (**right**). The sharp peak at 2.50 ppm is due to the (CD_3_)_2_SO used as a solvent.

The functionalized PK30IM and the derived PK30IMq, PK30IMq_80, PK30IMq_90, and PK30IMq_95 membranes were then characterized by FTIR (Figure 2a). Inspection of the spectra reveals that the intensity of the band at 1704 cm^−1^ ascribed to the PK carbonyl groups decreased in agreement with the functionalization with 1-(3-aminopropyl)imidazole. The membranes and the PK30IM showed similar FTIR absorptions, with the presence of bands at around 3108 cm^−1^, 1503 cm^−1^, and 714 cm^−1^ related to the stretching vibration of the C=C bonds, bending of the =C-H bonds, and C-C vibration of the pyrrole groups, respectively [25]. In addition, the membranes showed a low-intensity peak at around 1680 cm^−1^ that was assigned to the quaternized imidazole group. The chemical structures of PK30 and PK30IM were also characterized in terms of ^1^H-NMR (Figure 2b), which confirmed the success of the Paal–Knorr reaction. Three peaks at 7.65, 7.21, and 6.89 ppm were attributed to the imidazole ring [26], whereas that at 5.75 ppm was ascribed to the presence of the pyrrole ring [18,25]. The peak at 2.77 ppm was instead assigned to the -CH_2_ protons of the polymer backbone. 

**Figure 3:**
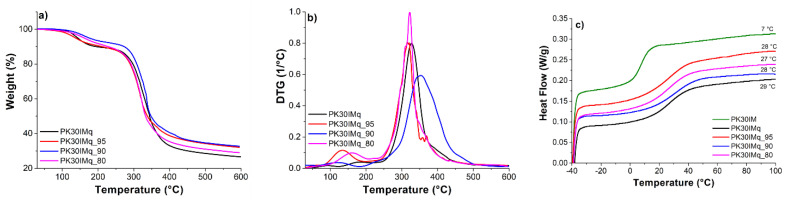
(**a**) The thermogravimetric curves and (**b**) their derivatives of PK30IMq and PK30IMq_n (n = 95, 90, and 80). (**c**) Second heating DSC scans of the AEMs, with the midpoint of the curves for all of the samples, including PK30IM.

We also evaluated the thermal behavior of the membranes by thermogravimetric analysis (TGA) and differential scanning calorimetry (DSC) (Figure 3a,b). Inspection of the TGA curves showed that the membranes had a strong ability to bind water; thus, the weight loss that occurred at around 100 °C was attributed to the evaporation of water. In addition, they showed an onset decomposition temperature at around 133–150 °C, with the 10% weight loss attributed to the degradation of quaternary ammonium groups. The second degradation step at 315–350 °C, with an overall weight loss of 55%, was attributed to the polyketone backbone. Notably, PK-based membranes exhibited good thermal stability and met the temperature requirements for applications as AEMs for fuel cells and electrolyzers [27]. DSC analysis showed that all of the samples were amorphous, and the T_g_ of PKIM increased from 7 °C to 27–29 °C after quaternization with 1,4-iodobutane and/or 1-iodobutane, in agreement with the restricted degree of freedom caused by the interaction between the macromolecular chains and the crosslinking degree (Figure 3c). 

**Figure 4:**
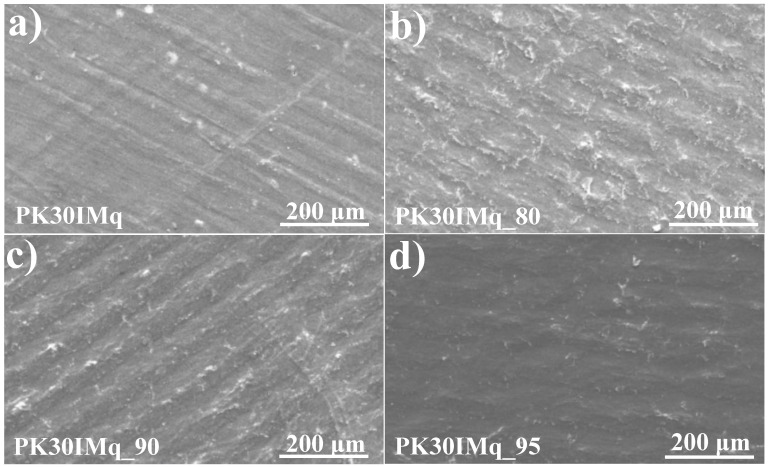
SEM images of (**a**) PK30IMq, (**b**) PK30IMq_80, (**c**) PK30IMq_90, and (**d**) PK30IMq_95.

Figure 4 shows the SEM micrographs of the sample PK30IMq and the membranes PK30IMq_n (n = 80, 90, and 95). Inspection of the micrographs showed the presence of a rough surface, with microscopic regular figures caused by the surface texture of the Petri dish used for the sample preparation. Regardless, no holes or cracks were present on the membrane surfaces, suggesting that the quaternization process did not affect the integrity and homogeneity of the material. The iodine content of the membranes was determined by energy-dispersive X-ray spectroscopy analysis, and the results are reported in Table 2. The membranes showed an iodine content between 20 and 31 wt.%, proving the effectiveness of the quaternization step. The variability of the iodine content in the samples could be attributed to an uneven distribution of the quaternized imidazole moieties, which may have caused the iodine contents to differ at the microscale. 

**Figure 5:**
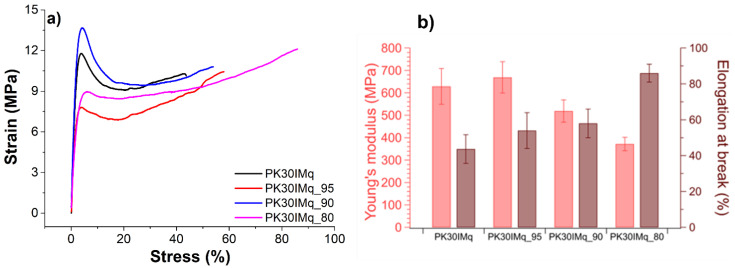
(**a**) The mechanical properties of the PK30IMq_n (n = 80, 90, and 95). (**b**) Their tensile strength (MPa) and elongation at break (%).

The mechanical characterization was carried out to assess the mechanical resistance of the membrane under uniaxial stress. Figure 5 shows the membrane’s stress–strain curves recorded on samples with similar thickness (around 160 μm), and the derived Young’s modulus and elongation at break (%) are summarized in Table 3. As expected, the least-crosslinked membrane (PK30IMq_80) showed the lowest Young’s modulus (372 ± 30 MPa) and the highest elongation at break (86 ± 5 %) compared to the other membranes, due to the use of the highest amount of 1-iodobutane as the quaternizing agent. As expected, the use of 1-iodobutane for the quaternization of the imidazole groups provided a less-crosslinked material, whose reduced rigidity might result in increased membrane durability in the alkaline cell.

### 3.2. Water Uptake and Ion-Exchange Capacity 

Water uptake (WU) and ion-exchange capacity (IEC) measurements were performed on the sample PK30IMq and the membranes PK30IMq_n (n = 80, 90, 95) to evaluate the AEMs with ex situ characterization techniques. Table 4 shows the WU data of the membranes, indicating how the membrane mass changes when exposed to water [10]. The samples did not show significant differences in WU, and the data were higher than those of similar membranes reported in the literature of about 15%, possibly due to the lower molecular weight of the starting PK utilized in this study [25,26]. Nevertheless, a maximum WU of about 38% possibly suggests good mechanical stability of the AEM during the working operations in the cell. The IEC is a measure of the number of ions exchanged per dried membrane weight [10], and the calculated values are reported in Table 4. As expected, all of the membranes showed similar IEC, in agreement with the WU experiments and literature data, and sometimes even higher than those measured at room temperature [25,26]. 

### 3.3. Electrolytic Cell Tests

**Figure 6:**
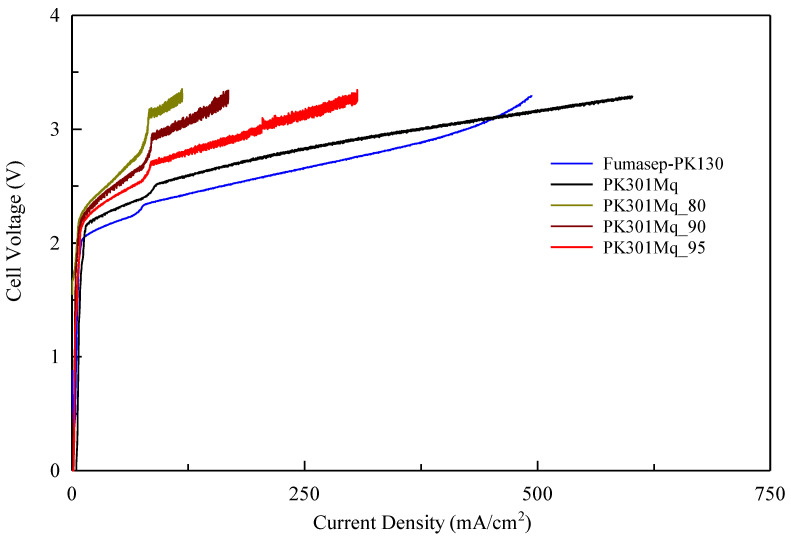
Polarization curves measured during water electrolysis using the ENEA electrolytic test cell.

The samples were activated before the electrolytic analysis with a 0.5 M KOH solution for 24 h, washed, and kept in distilled water at 25 °C. With the aim of obtaining results associated with the membrane only, no powder catalyst was used. The measurements were carried out from 0 to 800 mA/cm^2^, but they were automatically stopped when they exceeded 3.2 V, since commercial electrolyte cells usually work in a range of 1.85–2.05 V [19]. Figure 6 and Table 5 show the results obtained through the electrolytic cell tests at room temperature and atmospheric pressure [28]. PK30IMq showed the lowest resistance of 0.65 Ω and the highest ionic conductivity of 9.69 mS/cm, compared to the less-crosslinked membranes, and in agreement with the determined WU and IEC values. It is worth noting that the ionic conductivity of PK30IMq was comparable with that of a benchmark AEM (i.e., 9.66 mS/cm, Fumasep-PK-130) measured with the same experimental setup. However, one of the problems encountered was the chemical fragility of the PK30IMq and PK30IMq_95 membranes, since their performance was reduced considerably after 20 h in the cell, due to the membrane degradation on the anode (see Supplementary Materials, Table S2). The PK30IMq_90 and PK30IMq_80 membranes did not present this problem, but their performances were considerably lower. The measured flow of H_2_ produced during the electrolysis test (in cm^3^ per hour) is reported in Table S3 and was consistent with the performances of the prepared membranes. 

The lower ionic conductivity of the less-crosslinked systems was unexpected, since no variations were assumed considering the similar IEC values. Most probably, these discrepancies could be addressed by the reproducibility of the quaternization procedure when a mixture of iodides is used. However, the results obtained are promising and suggest continuing the research on these materials, which—through appropriate changes in the chemical composition—could easily meet the requirements for their use in electrolytic cells [27].

## 4. Conclusions

AEMs were successfully prepared by chemically modifying PK30 with 1-(3-aminopropyl)imidazole, followed by its quaternization/crosslinking with 1,4-iodobutane or its mixture with different amounts (5–20%) of 1-iodobutane. Spectroscopic investigations confirmed the successful modification of the PK backbone and the presence of the pyrrole and imidazole moieties, with a conversion (C_CO_ %) of 33%. The AEMs were found to be thermally stable up to 130 °C and 100% amorphous, with a T_g_ of 27–29 °C, depending on the crosslinking degree. All of the prepared membranes were homogeneous and had iodine contents between 20 and 31 wt.%. The mechanical properties depended on the crosslinking degree of the membrane, with a minimum Young’s modulus of 372 ± 30 MPa and a maximum of 86 ± 5 % for the elongation at break for the PK30IMq_80 membrane. The ionic conductivity at 25 °C reached a maximum value of 9.69 mS/cm for the PK30IMq membrane, along with a WU of 38 ± 2 % and IEC of 2.22 ± 0.02 meq/g, showing the excellent potential of these systems as AEMs. However, PK30IMq showed fast degradation during its work in the electrolytic cell, and future efforts will be directed toward enhancing the structural stability of these PK-based AEM systems. 

## Figures and Tables

**Table 1:** Amounts (in mg) of 1,4-diiodiobutane and/or 1-iodiobutane, and mixture composition (%), used for the different membranes.

Sample	PK30IM mg	1,4-Diiodiobutane mg (%)	1-Iodiobutane mg (%)
PK30IMq	500	116 (100)	0 (0)
PK30IMq_95	500	92 (95)	27 (5)
PK30IMq_90	500	104 (90)	13 (10)
PK30IMq_80	500	110 (80)	6 (20)

**Table 2:** Iodine contents determined by the EDS analysis of the samples PK30IMq and PK30IMq_n (n = 80, 90, and 95).

Sample	Iodine Content (wt.%)
PK30IMq	25
PK30IMq_80	20
PK30IMq_90	22
PK30IMq_95	31

**Table 3:** Young’s modulus (MPa) and elongation at break (%) values for the samples PK30IMq and PK30IMq_n (n = 95, 90, and 80).

Sample	Young’s Modulus (MPa)	Elongation at Break (%)
PK30IMq	629 ± 80	44 ± 8
PK30IMq_95	372 ± 30	86 ± 5
PK30IMq_90	669 ± 70	54 ± 10
PK30IMq_80	519 ± 50	58 ± 8

**Table 4:** Water uptake and ion-exchange capacity of the samples PK30IMq and PK30IMq_n (n = 95, 90, and 80).

Sample	WU (%)	IEC (meq/g)
PK30IMq	38 *±* 2	2.22 *±* 0.02
PK30IMq_80	33 *±* 5	1.92 *±* 0.01
PK30IMq_90	35 *±* 1	2.1 *±* 0.3
PK30IMq_95	37 *±* 2	1.9 *±* 0.2

**Table 5:** Resistance, ASR (area-specific resistance), and ionic conductivity of the Fumasep-PK-130, PK30IMq, and PK30IMq_KOH_n (n = 95, 90, and 80) AEMs.

Sample	Resistance (Ω)	ASR (Ω*cm^2^)	Ionic Conductivity (mS/cm)
PK30IMq	0.65	1.30	9.69
PK30IMq_80	2.88	5.76	3.47
PK30IMq_90	1.82	3.64	3.76
PK30IMq_95	1.57	3.14	3.28
Fumasep-PK-130	0.71	1.42	9.66

## Data Availability

The data presented in this study are available upon request from the corresponding author.

## Supplementary Materials

The following supporting information can be downloaded at: https://www.mdpi.com/article/10.3390/polym15092027/s1, Figure S1: Comparison of the thermogravimetric curves of the samples PK30 (black) and PK30IM (red); Figure S2: DSC trace of the second heating of the sample PK30; Figure S3: Experimental cell for electrochemical measurement and photo of a PK-based membrane prepared in this study; Table S1: Elemental composition of the PK30IM sample; Table S2: Voltage cell data in the first 20 h of electrolysis, and the relative efficiency; Table S3: Measured flow during the electrolysis test.
