# Supplementary material for: Polyketone-Based Anion-Exchange Membranes for Alkaline Water Electrolysis
Source: Polymers (Basel). 2023 Apr 25;15(9):2027. doi: 10.3390/polym15092027 (PMC10180749; doi:10.3390/polym15092027)
Supplement: Supplementary file 1 [file polymers-15-02027-s001.zip › polymers-2302463-supplementary.pdf]

polymers

Supplementary Materials: Polyketone-based anion-exchange membranes for alkaline water electrolysis

Ottavia Racchi, Rebecca Baldassari, Esteban Araya-Hermosilla, Virgilio Mattoli, Pierpaolo Minei, Alfonso Pozio and Andrea Pucci

Table S1. Composition of PK30IM sample by elemental analysis.

Sample name	C %	H %	N %	Cco%
Theoretical PK30IM	70.35	8.13	10.05	40
PK30IM	66.00	7.79	8.62	33

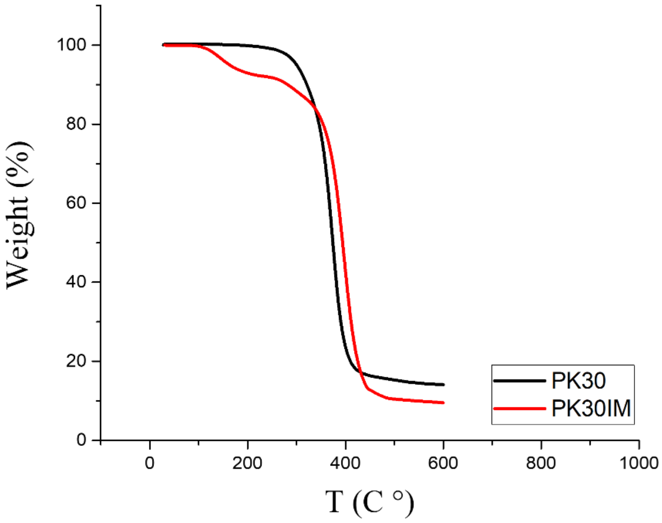

Figure S1. Comparison of the thermogravimetric curves of the sample PK30 (black) and PK30IM (red).

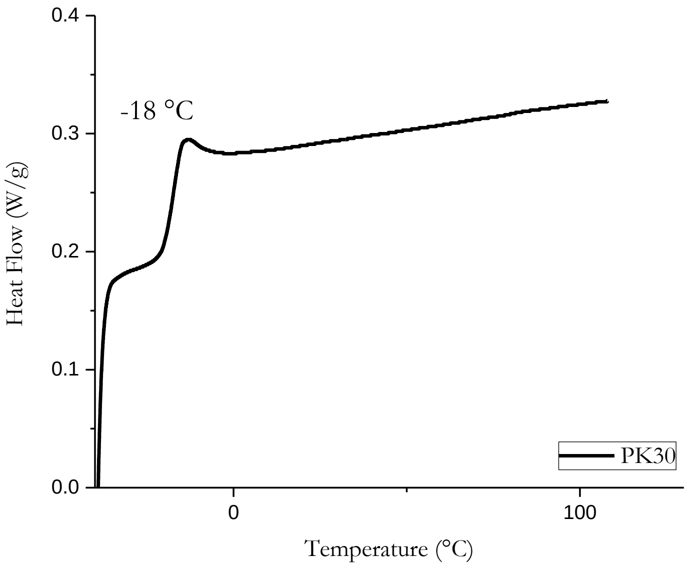

Figure S2. DSC trace of the second heating of the sample PK30.

Table S2. Voltage cell data in the first 20 h of electrolysis and the relative efficiency. At 50 mA/cm^2 the average voltages were:

Sample name	V (efficiency, %)
PK30IMq	2.06 Volt (71.8%)
PK30IMq_95	2.05 Volt (72.2%)
PK30IMq_90	2.30 Volt (64.3%)
PK30IMq_80	2.40 Volt (61.7%)

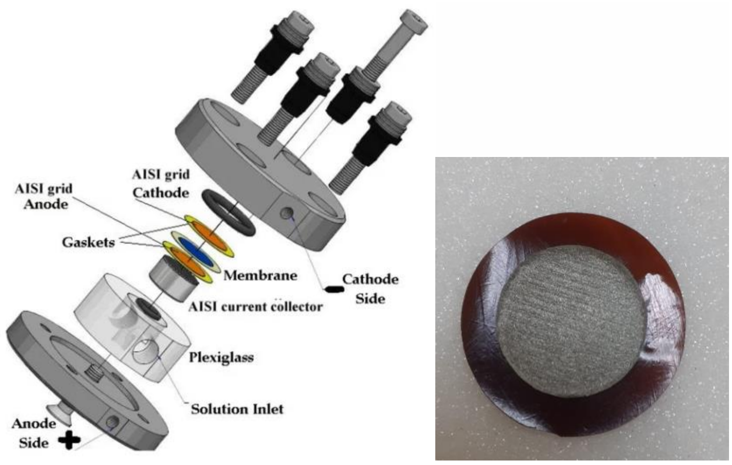

Figure S3. Experimental cell for electrochemical measurement and photo of a PK-based membrane prepared in this study.

Table S3. Measured flow during electrolysis test (in cm^3 per hour).

Sample	H_2 Flow (cm^3/h)
PK30IMq	209
PK30IMq_80	38
PK30IMq_90	55
PK30IMq_95	100
Fumasep-PK130	208
